# Latitude, Birth Date, and Allergy

**DOI:** 10.1371/journal.pmed.0020294

**Published:** 2005-10-04

**Authors:** Matthias Wjst, Shyamali Dharmage, Elisabeth André, Dan Norback, Chantal Raherison, Simona Villani, Jure Manfreda, Jordi Sunyer, Deborah Jarvis, Peter Burney, Cecilie Svanes

**Affiliations:** **1**Gruppe Molekulare Epidemiologie, Institut für Epidemiologie, Forschungszentrum für Umwelt und Gesundheit, Munich, Germany,; **2**Department of Public Health, School of Population Health, The University of Melbourne, Carlton, Victoria, Australia,; **3**Department of Medical Science, Occupational and Environmental Medicine, Uppsala University, Uppsala, Sweden,; **4**Service des Maladies Respiratoires, Centre François Magendie, Hôpital du Haut-Lévêque, Pessac, France,; **5**Università egli Studi di Pavia, Dip. di Scienze Sanitarie Applicate e Psicocomportamentali, Pavia, Italy,; **6**University of Manitoba, Department of Medicine, Winnipeg, Manitoba, Canada,; **7**Respiratory and Environmental Health Research Unit, Municipal Institute of Medical Research, Barcelona, Spain,; **8**Department of Public Health Sciences, Capital House, London, Great Britain,; **9**Det medisinske fakultet, Lungemedisin, Universitet I Bergen, Bergen, Norway,; National Heart and Lung InstituteUnited Kingdom

## Abstract

**Background:**

The space and time distribution of risk factors for allergic diseases may provide insights into disease mechanisms. Allergy is believed to vary by month of birth, but multinational studies taking into account latitude have not been conducted.

**Methods and Findings:**

A questionnaire was distributed in 54 centres to a representative sample of 20- to 44-y-old men and women mainly in Europe but also including regions in North Africa, India, North America, Australia, and New Zealand. Data from 200,682 participants were analyzed. The median prevalence of allergic rhinitis was 22%, with a substantial variation across centres. Overall, allergic rhinitis decreased with geographical latitude, but there were many exceptions. No increase in prevalence during certain winters could be observed. Also, no altered risk by birth month was found, except borderline reduced risks in September and October. Effect estimates obtained by a multivariate analysis of total and specific IgE values in 18,085 individuals also excluded major birth month effects and confirmed the independent effect of language grouping.

**Conclusion:**

Neither time point of first exposure to certain allergens nor early infections during winter months seems to be a major factor for adult allergy. Although there might be effects of climate or environmental UV exposure by latitude, influences within language groups seem to be more important, reflecting so far unknown genetic or cultural risk factors.

## Abstract 2

A large international survey of risk factors associated with allergy refutes previous work suggesting that allergy varies by month of birth

## Introduction

Allergy prevalence has been on the rise in many countries, while causal risk factors are still unknown [1]. The spatial and temporal distribution of risk factors may offer an insight into the mechanism of disease.

Birth month has been claimed to be associated with allergy. More than half of the studies summarized in our first analysis of birth month and allergy in 1992 [2] showed a positive association of month of birth with various allergy outcomes [3–19]. A few studies missed at that time, as well as most consecutive studies [20–36] do not show a consistent relationship.

Birth month has been used as a proxy for early allergen exposure but may also be associated with upper respiratory infections during certain winter months. At least in Europe, exposure to outdoor allergens is expected to occur in annually fixed flowering intervals, while episodes of respiratory infections are encountered in autumn and winter months with variation between years. Any autumn or winter season of birth effect could give further support the hygiene hypothesis [37] that postulates a reduction of natural infection, which is responsible for an over-reactive immune system, finally leading to allergy.

Geographical latitude so far has been associated with different diseases such as Crohn disease [38] or type I diabetes [39] but only sporadically with allergy [40]. Latitude is usually described as a proxy for UV solar exposure, as radiation reaching the earth's surface varies inversely with latitude. It may also reflect climatic differences responsible for different pollen seasons, as well as different building construction. In addition, many other factors are associated with geographical latitude in Europe, such as genetic influences or cultural differences in raising children.

The aim of this analysis was, therefore, to further delineate latitude and birth date effects on the prevalence of allergy defined by markers such as allergic rhinitis (AR), sensitization to grass or dust, and total IgE levels.

## Methods

### Sample

The methods for the European Community Respiratory Health Study (ECRHS) I were published earlier [41], with protocols and questionnaires available from the study Web site (http://www.ecrhs.org). Briefly, ECRHS I participating centres were each selected from an area defined by pre-existing administrative boundaries, with a population of at least 150,000 people. An up-to-date sampling frame was used to randomly select at least 1,500 men and 1,500 women aged 20 to 44 y. All individuals were sent a questionnaire enquiring about respiratory symptoms and attacks of asthma in the last 12 mo, current use of asthma medication, and nasal allergies including hayfever (ECRHS I screening). This sample consists of 54 centres with 200,682 participants. For this analysis, the study centre Aarhus and part of Erfurt probands were excluded because of unreliable birth dates, in addition to all individuals with wrong or missing birth dates and all born on the 29th of February. Also, only birth years from 1945 until 1973 were included, as all other birth years did not have enough observations to be reliable. The final dataset included 186,723 individuals (Table 1). The main outcome variable in this dataset was the response to the question “Do you have any nasal allergies including ‘hayfever'?” Given 16,000 exposed persons in a single month compared to 16,000 born in a reference month with an assumed disease prevalence of 22% and a given α of 0.05, an increase of 1% in the exposed group would have been found with a power of 57% in a two-tailed test, while an increase of 2% would have been found with a power of 99%.

**Table 1:**
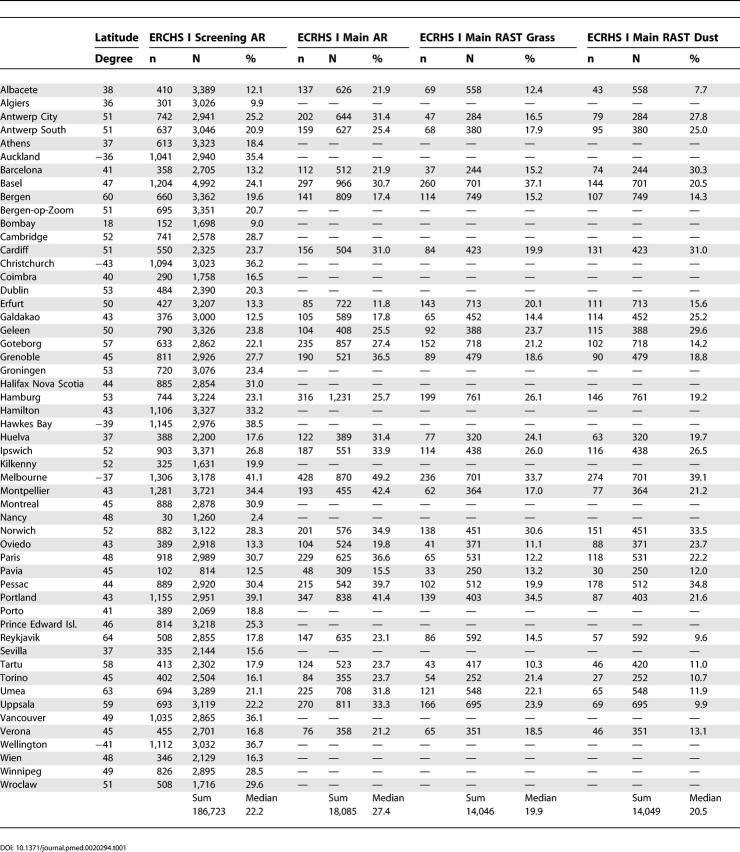
Lifetime Prevalence of AR and Prevalence of Positive RAST Values by Centre in ECRHS I Screening

A random sample of these individuals was selected to take part in the full study (ECRHS I main, in joint papers denominated stage II), during which they were invited to visit a local testing centre, answer a more detailed questionnaire, provide a blood sample for measurement of specific IgE and total IgE, perform baseline spirometry, and undergo bronchial challenge with methacholine. Informed consent was obtained from 60% of invited participants [42]. All study protocols were approved by the local ethics committees. Blood samples were all handled in a similar manner and analysed in a central laboratory (Pharmacia, Uppsala, Sweden). Briefly, blood samples were centrifuged and serum stored at −20 °C until total IgE and specific IgE analysis (Radio AllergoSorbent Test [RAST]), of which only mixed grass and house dust mite were used in this study. RAST class 1 or greater was considered as a positive antibody test. All total IgE values were log-transformed and tested on a continuous scale. Numbers under analysis are given in Table 1.

### Exposure

The following birth-related variables were defined from the questionnaire: sinus(day of year [1,...,365]); day of month (1,…,31); day of week (1 = Sunday,.., 7 = Saturday); month of year (1 = Jan,…,12 = Dec); season (1 = Dec/Jan/Feb, …, 4 = Sep/Oct/Nov); annual season (1/1945, …, 4/1973) and year of birth (1945,…,1973). Reported in this study are only month, year, and quarter of birth year, as none of the other variables showed any additional information.

**Figure 1:**
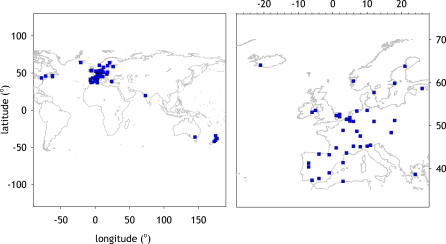
World Map and European Map of Study Centres

**Figure 2:**
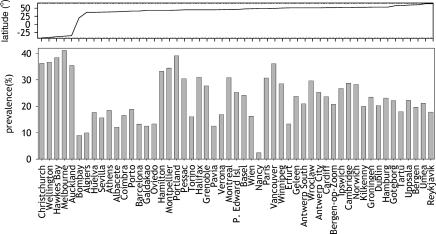
Prevalence of AR in ECRHS I Screening by Centre Centres are sorted by increasing geographical latitudes from left to right.

Geographical latitude was obtained from the route planning software Mapsonic (http://www.viamichelin.com, where GPS degrees were obtained for a random inner city point and were included as a continuous variable as well as dichotomized into quartile groups. Non-European locations were taken from The World Gazetteer (http://www.world-gazetteer.com) or Encarta (http://encarta.msn.com). For Figures 1 and 2, the original latitude values are used, while for all regression models only the absolute latitude values are taken. If primary language was not self evident, the CIA's World Factbook (http://www.cia.gov/cia/publications/factbook/fields/2098.html) was used as a reference. The city of Montreal was classified as English, although English/French bilingual questionnaires were used. Additional data included in this study are obtained from http://rimmer.ngdc.noaa.gov/mgg/coast/getcoast.html, http://www.polleninfo.org, and http://www.dssresearch.com/toolkit/spcalc/power_p2.asp.

### Analysis

In an initial step, Trellis barcharts and boxplots [43] were created for all date and latitude variables in the ECRHS I screening sample and contingency tables analysed using global χ^2^ tests. In a second step this analysis was repeated in ECRHS I main study for AR, total IgE, RAST grass, and RAST house dust. Heterogeneity across centres was assessed using meta-analysis with latitude included as a random effect [44]. Next, generalised linear equations were fitted with a binomial outcome for categorical and Gaussian outcome for IgE values. Analyses were conducted for single risk factors alone (Table 2), followed by the joint inclusion of all factors (Table 3). Open-source R software 2.0.1 was used for all analyses (http://www.r-project.org).

**Table 2:**
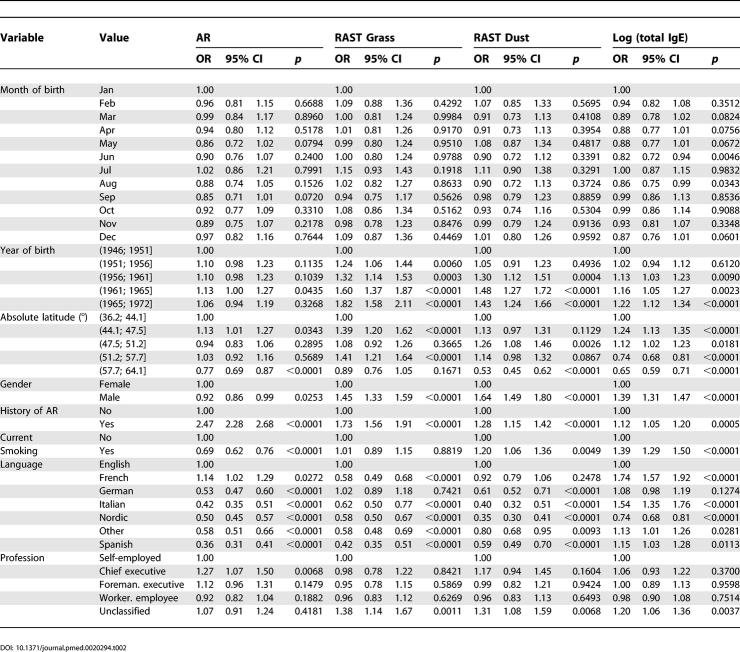
Crude Risk for AR, Total IgE, and Sensitization against Grass or House Dust Mite in ECRHS I Main Study

**Table 3:**
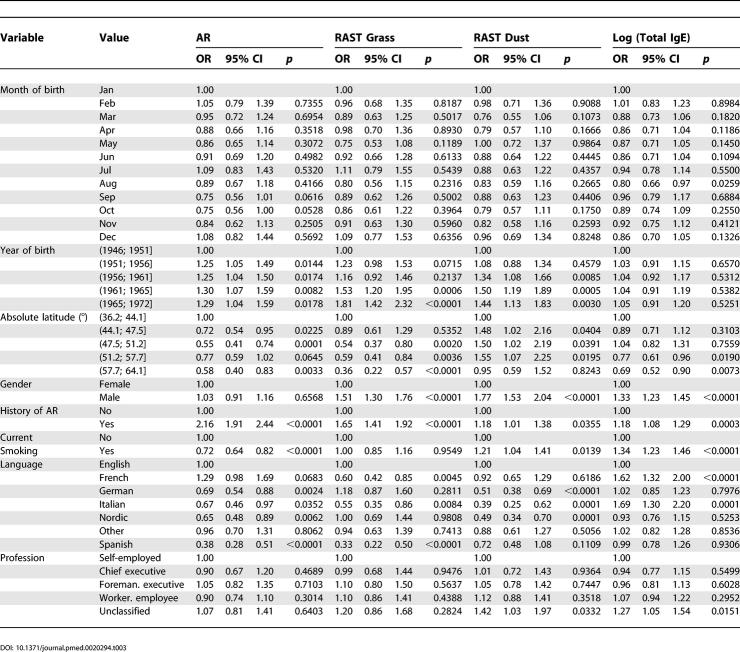
Adjusted Risk for AR, Total IgE, and Sensitization against Grass or House Dust Mite in ECRHS I Main Study

## Results

A history of AR was reported in the screening questionnaire with a range from 2.4% in Nancy to 41.1% in Melbourne (Table 1). Participants reported on median a prevalence of 22.2%, while the prevalence in ECRHS I main was slightly higher, reaching 27.4% (Table 1).

There was a substantial variation between centres in the frequency of sensitization against mixed grass allergen, ranging from 10.3% in Tartu up to 37.1% in Basel. Dust mite sensitization was high in Melbourne, Norwich, and Pessac, and low in Albacete, Reykjavik, and Uppsala.

All centres (Figure 1) were then ordered by geographical latitude (Figure 2). Latitudes covered in Europe include +37 ° (Huelva in Spain) up to +64 ° (Reykjavik in Iceland). Overall, AR decreased with geographical latitude, but there were many exceptions (Figures S1–S12).

The analysis of birth dates in ECRHS I screening did not show any significant effect, neither for day of year, day of month, nor day of week. Crude risks by birth month in ECRHS I main were also not increased, while in the adjusted analysis only September (OR 0.75; 0.56–1.01) and October (OR 0.75; 0.56–1.00) reached borderline significant reduced risks compared to birth month January.

When tested for heterogeneity according to centre, only birth month May showed significant centre differences (*p* = 0.021). There may be even more altered risks at the single centre level (like Huelva in January, Montpellier in February, etc.; see Figures S9 for ECRHS I screening and Figure S20 for ECRHS I main), but the overall number of associations does not seem to exceed the number expected by chance.

AR prevalence was high even in the earliest birth cohorts (Figures S1–S11 for ECRHS I screening and Figures S12–S22 for ECRHS I main). Analysing birth date in increasing 3-mo intervals did not reveal any major spike that could be attributed to a seasonal influence of winter months or the known influenza A pandemic years in 1957 and 1968 [45].

Finally, effects were analysed by generalized linear regression models in ECRHS I main (Table 2) that also controlled for confounding effects (Table 3). Factors with significant influence on AR in the adjusted model are a history of AR in the parents, current smoking, latitude, and language. There was also a significant effect by year of birth, but none by month of birth except for borderline reduced risks by being born in September or October. Latitude was negatively associated with AR (overall OR 0.85 per ten-degree increase, *p* < 0.0001 if included as a continuous variable).

Language appeared to be an independent factor from latitude and was in some instances an even stronger predictor for AR (Table 3). Language group affects not only AR and specific sensitization to all tested allergens, but also total IgE values. The most prominent effect compared to English language was seen for the Spanish language.

While risks for total and specific IgE were usually concordant, risks dissociated in the Italian group (OR for IgE grass 0.55, but 1.69 for total IgE), raising the question of further modifying factors of total IgE in Italy. AR and grass specific IgE were also discordant in the French centres, raising questions about labelling of AR in France (or the specificity of the tested allergens for AR there).

## Discussion

We describe a high prevalence of AR with a substantial variation across centres but do not find any major risk by being born in a particular month or during a particular season.

The main advantage of this study is the use of standardized interviews and identical laboratory methods, which leads to the conclusion that the geographical differences are real and not an artefact of non-comparable methods [46]. There is also less concern about non-response, since non-responder differences in the main outcome variables were relatively small [46]. Another benefit is the power of the study due to the high number of participants.

Previous studies of birth months showed mixed results. Due to the different definitions and outcomes used, studies can roughly be grouped into those with a positive [5–7,9,10,12–19,21,24,26,29,30,32–36], negative [3,4,8,27,47], or even unclear outcome [20,22,23,28,31]. Without applying formal criteria of a meta-analysis, these studies are difficult to sort. Some are case-only studies, others depend on cross-sectional data, and only a few are cohort studies. A detailed knowledge of local circumstances would be necessary to integrate all results into a larger framework. Also, this study agrees that there might be relevant birth month effects in single centres, but it questions any global effect.

Taking into account publication bias, it may be understandable that in published studies the positive results outnumber negative ones. Most of the previous studies showed an association with allergic sensitization (and not so often with AR), which may indicate subclinical effects that may gain importance only when occurring in combination with additional risk factors. One of the main advantages of our study—the standardized allergen test protocol—might be a disadvantage where the effects of local allergens might have been missed. An exposure matrix constructed by flowering season in all participating European centres did not result in a different risk estimate (unpublished data; see Materials and Methods).

A further difference in comparison with many previous studies is the higher age of our study participants. It may be possible that more marked symptoms exist in children, that are being lost in adulthood. We are sharing, however, a methodological problem with most previous studies, as we are assuming that current residence is identical with residence of birth, which might not always be true. For those participating in our main study, migration was limited [48] and is probably not leading to a distortion of study results.

Another methodological restriction may be the use of self-reported “hayfever.” This term might be used in a different way across Europe, and there might be secular changes in the labelling. The more or less negative finding for any particular birth month (together with the results for specific IgE against grass) in our study, however, is rather consistent across centres. It is possible that reporting of AR during “symptom” months might be increased, but there is no indication that any differential reporting is also associated with birth date. It is therefore unlikely that specific allergen exposure outdoors directly after birth has a major impact on the development of AR.

So far, no data confirm that allergen exposure has been increased in parallel with AR during the recent decades. This would be a rather likely explanation, as global climatic changes with warming may result in higher grass pollen exposure [49], and construction of better isolated houses may support dust mite growth. A study on peak allergen exposure, however, showed only an insignificant increased sensitization [50], and even allergen avoidance trials are far from being conclusive [51].

Any effect of winter month epidemics, in particular of the influenza A episodes in 1957 and 1968 [45], could not be shown. An increasing trend of asthma incidence by birth year has already been described in this study [52] and is now also found for AR. It may be emphasized that the prevalence in the cohorts born directly after World War II is already high and stable over all birth years. Unfortunately, time and cohort effects cannot be discriminated, but an effect by infection epidemics directly before or after birth is unlikely.

Data pertaining to the geographical distribution of allergic diseases are rare. The International Study of Asthma and Allergies in Childhood (ISAAC) compared the worldwide distribution of AR in children, which varied across centres from 0.8% to 14.9% in the 6- to 7-y-olds and from 1.4% to 39.7% in the 13- to 14-y-olds [53]. Although the prevalence in 13- to 14-y-old children was slightly lower, a direct comparison of 13 centres included in both ECRHS and ISAAC showed good agreement. A worldwide meta-analysis of ISAAC centres showed a negative association of latitude and symptoms of AR with a −0.05% decrease per degree (−0.11; 0.00) in 6- to 7-y-olds and with −0.09% (−0.18; −0.01) in 13- to 14-y-olds; effects were attributed to climatic differences such as indoor humidity or altitude [40]. A study in Australia also reported a negative association of latitude and asthma [39], while effects in this study are interpreted by UV solar radiation. This view may be also supported by a recent study of vitamin D supplementation and allergy [54].

AR decreased with geographical latitude, but it is unclear why the Spanish, Portuguese, and Greek centres make such an exception from this rule. Surprisingly, in another study the lowest serum vitamin D metabolite concentrations were seen in southern European countries, which could be explained by attitudes toward sunlight exposure [55].

The lowest prevalence of AR in the ISAAC was found in Eastern Europe and South and Central Asia, and—as in the ECRHS—a high prevalence was reported for English-speaking centres. It is intriguing that inclusion of preferential language into the multivariate model did not resolve the latitude effect, and even increased it.

Language may be a marker for genetic traits [56], and there is indeed a genetic heterogeneity in this study by language where a history of AR in the family does not countervail for the latitude effect. A risk factor operating within language borders therefore seems to be even more relevant than geographical latitude alone.

## Supporting Information

Figure S1 ECRHS I Screening AR Prevalence by Birth Quarter and Centre (298 KB PDF)

Figure S2 ECRHS I Screening AR Prevalence by Birth Quarter and Latitude/Longitude (105 KB PDF)

Figure S3 ECRHS I Screening AR Prevalence by Birth Quarter and Language (105 KB PDF)

Figure S4 ECRHS I Screening AR Prevalence by Birth Quarter and Sex (32 KB PDF)

Figure S5 ECRHS I Screening AR Prevalence by Month of Birth and Centre (87 KB PDF)

Figure S6 ECRHS I Screening AR Prevalence by Month of Birth and Latitude/Longitude (59 KB PDF)

Figure S7 ECRHS I Screening AR Prevalence by Month of Birth and Language (54 KB PDF)

Figure S8 ECRHS I Screening AR Prevalence by Month of Birth and Sex (48 KB PDF)

Figure S9 ECRHS I Screening AR Prevalence by Month and Year of Birth (66 KB PDF)

Figure S10 ECRHS I Screening AR Prevalence by Quarter and Month of Birth (66 KB PDF)

Figure S11 ECRHS I Screening AR Prevalence by Quarter and Month of Birth (51 KB PDF)

Figure S12 ECRHS I Main Median Log Total IgE by Birth Quarter and Centre (180 KB PDF)

Figure S13 ECRHS I Main Median Log Total IgE by Birth Quarter and Latitude/Longitude (120 KB PDF)

Figure S14 ECRHS I Main Median Log Total IgE by Birth Quarter and Language (85 KB PDF)

Figure S15 ECRHS I Main Median Log Total IgE by Birth Quarter and Sex (58 KB PDF)

Figure S16 ECRHS I Main Median Log Total IgE by Month of Birth and Centre (70 KB PDF)

Figure S17 ECRHS I Main Median Log Total IgE by Month of Birth and Latitude/Longitude (61 KB PDF)

Figure S18 ECRHS I Main Median Log Total IgE by Month of Birth and Language (53 KB PDF)

Figure S19 ECRHS I Main Median Log Total IgE by Month of Birth and Sex (47 KB PDF)

Figure S20 ECRHS I Main Median Log Total IgE by Month and Year of Birth (67 KB PDF)

Figure S21 ECRHS I Main Median Log Total IgE by Month and Quarter of Birth (66 KB PDF)

Figure S22 ECRHS I Main Median Log Total IgE by Absolute Latitude and Language (58 KB PDF)

**Patient Summary**

**Background**

Allergy is becoming more common in many parts of the world, but there is no satisfactory explanation for this, or for why the number of people with allergies varies so much between countries. If scientists knew more about where and when the risks of allergy are highest, it might help them understand more about the condition and its causes. Some research has suggested that the month in which one is born can affect one's risk of getting an allergy, although other studies have not found this. The month of birth would determine how old a baby was when it first encountered an allergy-causing substance (like pollen) or an infection (like a cold), and this might turn out to be important.

**What Did The Researchers Do?**

They looked at one type of allergy: allergic rhinitis, the inflammation of the membranes inside the nose that is usually called hayfever. They gave a questionnaire to patients in 54 regions, mainly in Europe. More than 200,000 people responded, and nearly a quarter of them had allergic rhinitis. Overall, the condition was found to be more common further away from the Equator, although there were some exceptions. However, the month of birth did not seem to make a difference in the likelihood of getting allergy. The researchers did find a variation in allergy rates according to which languages people spoke. They suggest that this means there are genetic and cultural factors involved in allergy risk.

**What Does This Mean?**

This study was larger and used more reliable methods than some earlier research, so we can be more confident of the conclusions. The mystery of why allergy is becoming more common has not been solved, but the researchers' conclusion that genetic and cultural factors are more important than geographical factors is an advance in our knowledge.

**More Information Online**

General information for suffers of allergy, including hayfever, may be found on these Web sites.

The American Academy of Family Physicians:

http://familydoctor.org/083.xml

The American Academy for Asthma, Allergy and Immunology:

http://www.aaaai.org

Allergy UK, formerly the British Allergy Foundation:

http://www.allergyuk.org/allergy_whatis.html

For the big picture on asthma worldwide, try the World Allergy Organization:

http://www.worldallergy.org

## Abbreviations

AR: allergic rhinitis
ECRHS: European Community Respiratory Health Study
ISAAC: International Study of Asthma and Allergies in Childhood
RAST: Radio AllergoSorbent Test
