# Supplementary material for: Latitude, Birth Date, and Allergy
Source: PLoS Med. 2005 Oct 4;2(10):e294. doi: 10.1371/journal.pmed.0020294 (PMC1240049; doi:10.1371/journal.pmed.0020294)

Figure O-1: AR prevalence by birth quarter and centre.

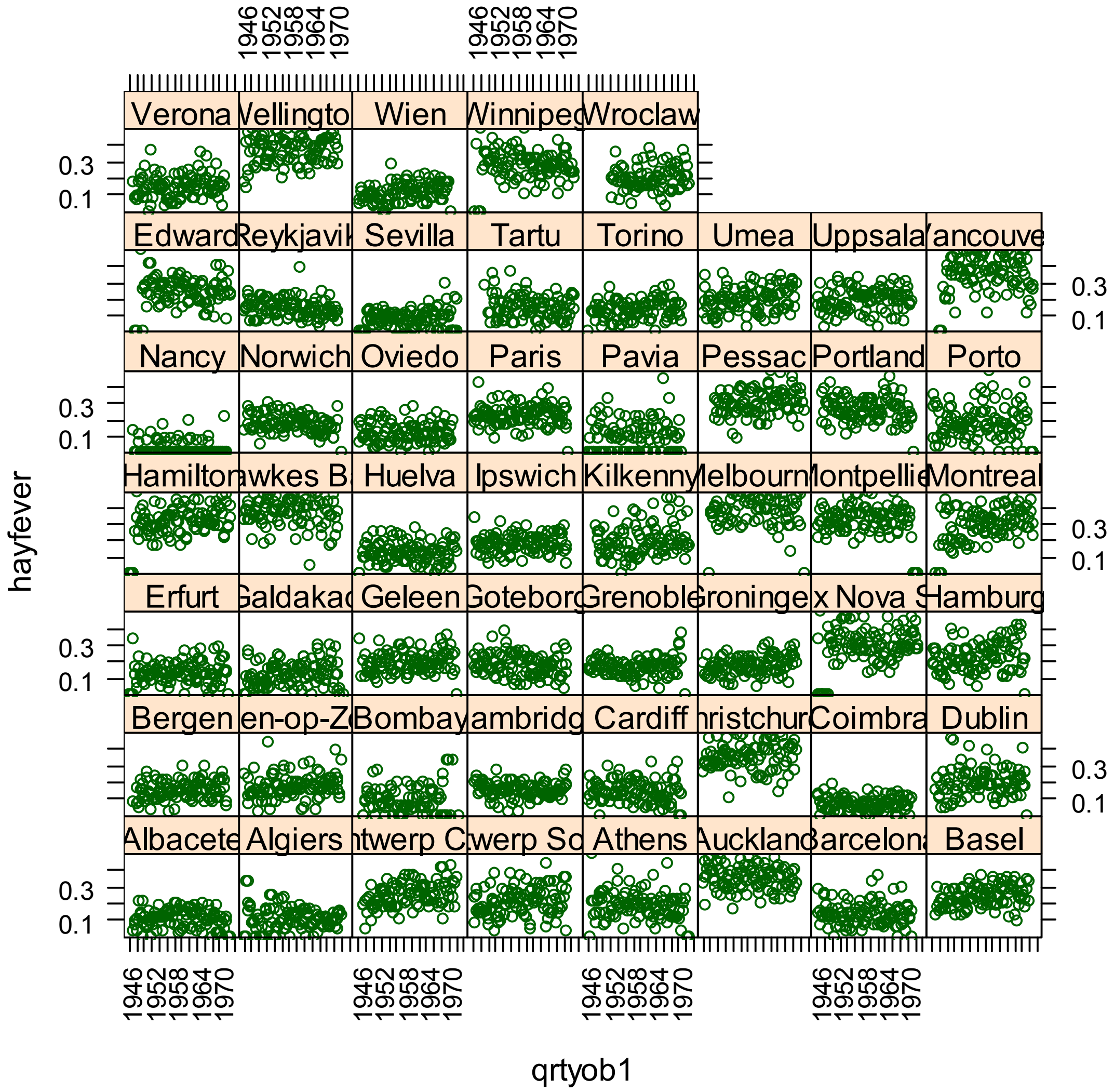

Supplement: Supplementary file 1 [file pmed.0020294.sg001.pdf]
