# Supplementary material for: Latitude, Birth Date, and Allergy
Source: PLoS Med. 2005 Oct 4;2(10):e294. doi: 10.1371/journal.pmed.0020294 (PMC1240049; doi:10.1371/journal.pmed.0020294)

Figure O-7: AR prevalence by month of birth and language.

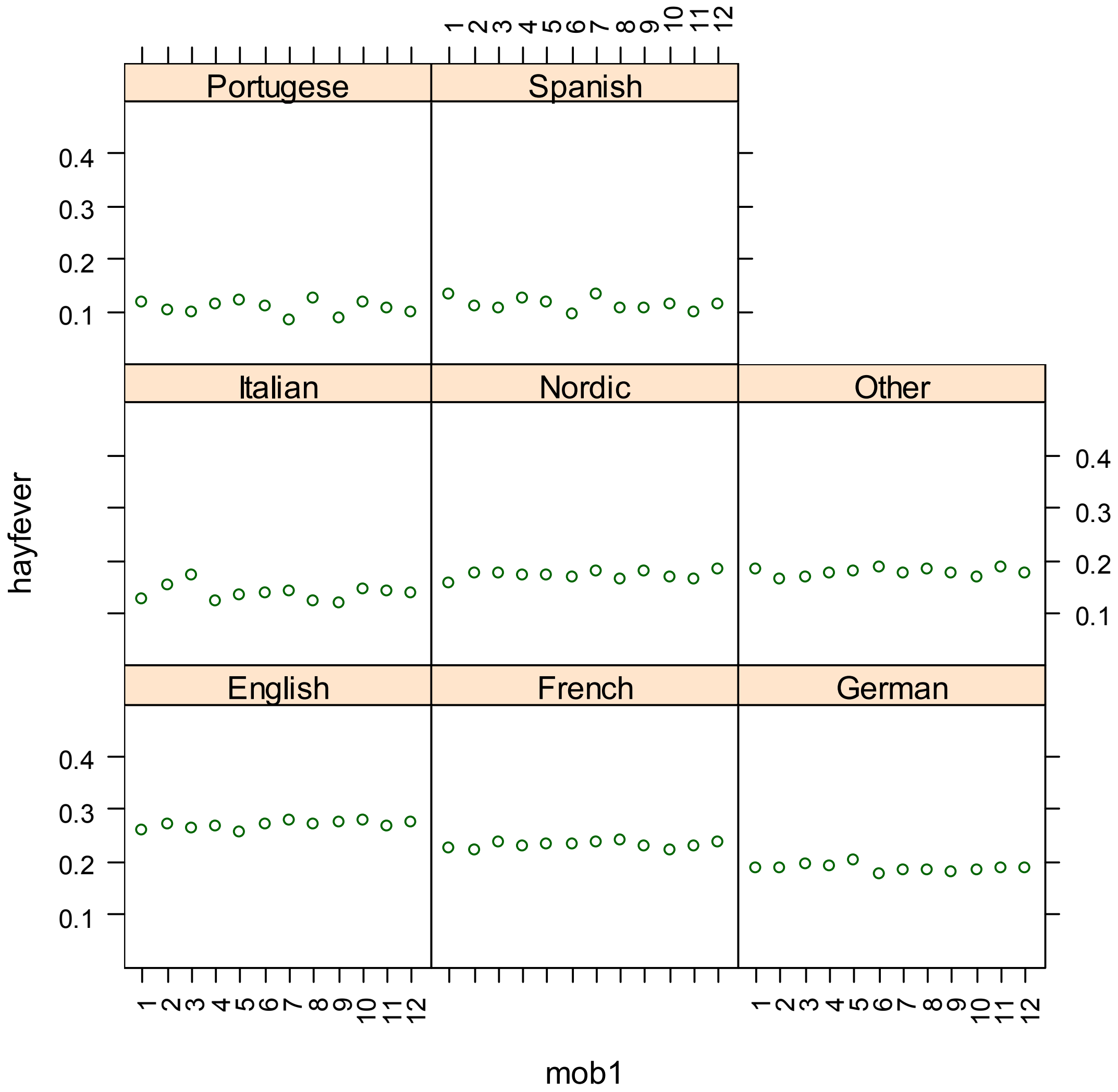

Supplement: Supplementary file 7 [file pmed.0020294.sg007.pdf]
