# Supplementary material for: Latitude, Birth Date, and Allergy
Source: PLoS Med. 2005 Oct 4;2(10):e294. doi: 10.1371/journal.pmed.0020294 (PMC1240049; doi:10.1371/journal.pmed.0020294)

Figure O-14: Median log total IgE by birth quarter and language.

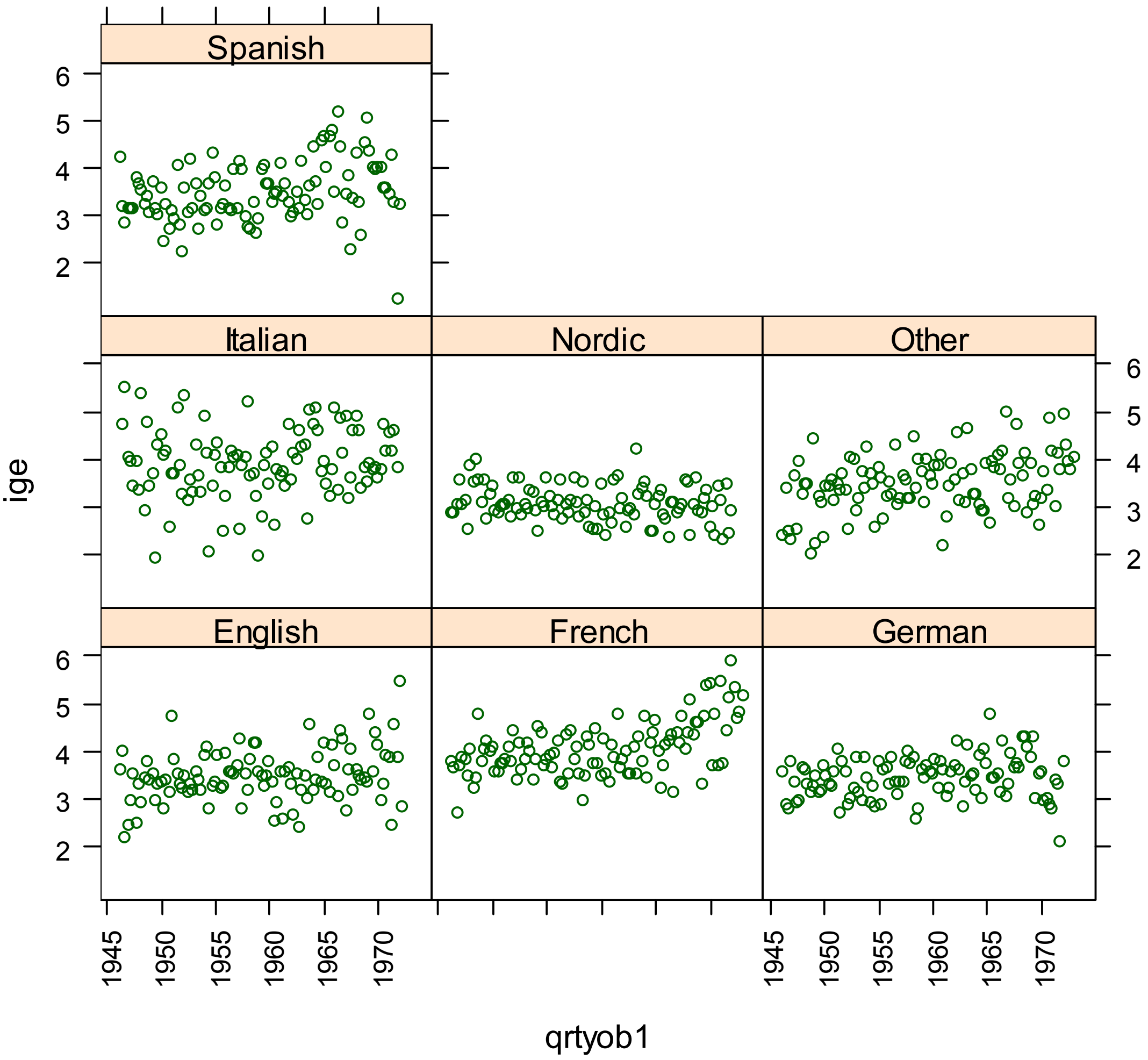

Supplement: Supplementary file 14 [file pmed.0020294.sg014.pdf]
